# Non-coding RNAs in Nervous System Development and Disease

**DOI:** 10.3389/fcell.2020.00273

**Published:** 2020-05-06

**Authors:** Beatrice Salvatori, Silvia Biscarini, Mariangela Morlando

**Affiliations:** ^1^Center for Life Nano Science@Sapienza, Istituto Italiano di Tecnologia, Rome, Italy; ^2^Department of Pharmaceutical Sciences, “Department of Excellence 2018-2022”, University of Perugia, Perugia, Italy

**Keywords:** ncRNAs, circRNAs, neurodegenerative diseases, biomarkers, therapeutics, neuronal development, synaptic activity

## Abstract

The rapid advance of RNA sequencing technologies contributed to a deep understanding of transcriptome composition and has allowed the discovery of a large number of non-coding RNAs (ncRNAs). The ability of these RNA molecules to be engaged in intricate and dynamic interactions with proteins and nucleic acids led to a great expansion of gene expression regulation mechanisms. By this matter, ncRNAs contribute to the increase in regulatory complexity that becomes highly specific between tissues and cell types. Among the ncRNAs, long non-coding RNAs (lncRNAs) and circular RNAs (circRNAs) are especially abundant in nervous system and have been shown to be implicated in its development, plasticity and aging as well as in neurological disorders. This review provides an overview of how these two diverse classes of ncRNAs control cellular processes during nervous system development, physiology, and disease conditions with particular emphasis on neurodegenerative disorders. The use of ncRNAs as biomarkers, tools, or targets for therapeutic intervention in neurodegeneration are also discussed.

## Introduction

The development and function of the nervous system relies on complex and well-orchestrated gene expression regulation occurring at multiple levels, from transcription to RNA processing, translation, and decay. New discoveries in transcriptomics, facilitated by technical advances in next generation sequencing and computational biology, have revealed the existence of a plethora of transcripts lacking coding potential but exerting an intense regulatory activity in a wide range of biological processes including neuronal development, differentiation, and function. These transcripts belong to the heterogeneous family of non-coding RNAs (ncRNAs) composed by several classes of genes, producing smaller molecules such as microRNAs (miRNAs), and longer transcripts that can be processed to form long non-coding RNAs (lncRNAs) and circular RNAs (circRNAs). Studies in the field of lncRNA and circRNA molecules have accelerated considerably during the last few years and major interest has grown in the use of these molecules as diagnostic and therapeutic targets in neurodegenerative diseases (Derrien et al., 2012; Salta and De Strooper, 2017).

LncRNAs are defined as a heterogeneous class of molecules longer than 200 nucleotides (nts) with no protein coding capacity. Their biogenesis is similar to that of mRNAs with RNA Polymerase II (RNAPII) engaging in their transcription. Nascent non-coding transcripts are often subjected to capping, canonical and alternative splicing as well as polyadenylation (Quinn and Chang, 2016). LncRNAs show tissue-specific expression, are generally present at low levels compared to the coding counterparts and show relatively low evolutionary conservation (Cabili et al., 2011; Derrien et al., 2012). Despite few examples, they generally lack an open reading frame (ORF) and influence gene expression at different levels through a variety of mechanisms of action, including recruitment of chromatin modifiers and transcription factors, regulation of three-dimensional chromatin folding, control of mRNA processing, translation and decay (Rinn and Chang, 2012; Fatica and Bozzoni, 2014).

In addition to these linear ncRNAs with distinct 5′ and 3′ ends, a group of circRNAs with covalently closed ends has recently gained attention. Initially discovered as plant viroids and hepatitis delta virus (Sanger et al., 1976; Kos et al., 1986) only in recent years the high-throughput RNA sequencing coupled with circRNA-specific bioinformatic algorithms revealed that 1000s of circRNA molecules are produced from a large fraction of genes in metazoans (human, mouse, zebrafish, worm, fruit fly) (Salzman et al., 2012; Jeck et al., 2013; Jeck and Sharpless, 2014; Salzman, 2014; Wang et al., 2014). Their biogenesis relies on a peculiar splicing reaction called back-splicing that joins a downstream 5′ splice site to an upstream 3′ splice site. Not much is known about their function, however, the few examples that have been characterized have revealed that circRNAs can potentially regulate gene expression both at transcriptional and post-transcriptional level (Kristensen et al., 2019).

Notably both lncRNAs and circRNAs are preferentially expressed in the nervous system and resulted to be dynamically regulated during neuronal development as well as in response to neuronal activity (Kim et al., 2010; Mercer et al., 2010; Derrien et al., 2012; Lipovich et al., 2012; Aprea et al., 2013; Rybak-Wolf et al., 2015; You et al., 2015; Biscarini et al., 2018). They also show highly restricted expression in various anatomic brain regions and cell structures (Mercer et al., 2008; Rybak-Wolf et al., 2015; You et al., 2015). These dynamics and region-specific expression patterns strongly suggest that lncRNAs and circRNAs may play key roles in nervous system development and function. Moreover, recent studies have also shown that, similar to protein coding genes, dysregulation of ncRNA molecules can affect proper nervous system development and function thus contributing to the onset and progression of neurological diseases (Shao and Chen, 2016; Wang et al., 2018a).

This review provides a comprehensive description of lncRNA and circRNA biogenesis and function highlighting their involvement in nervous system development and physiology. It also underlines the implication of ncRNA deregulation in diverse neurodegenerative disorders and ultimately how ncRNAs might serve as suitable diagnostic biomarkers and therapeutic targets.

## Long Non-Coding RNAs: Identification and Genomic Characterization

Analysis of transcriptomes through a high-resolution RNA sequencing (RNA-seq) is one of the most robust methodologies for the *de novo* identification of lncRNAs (Mortazavi et al., 2008).

**FIGURE 1:**
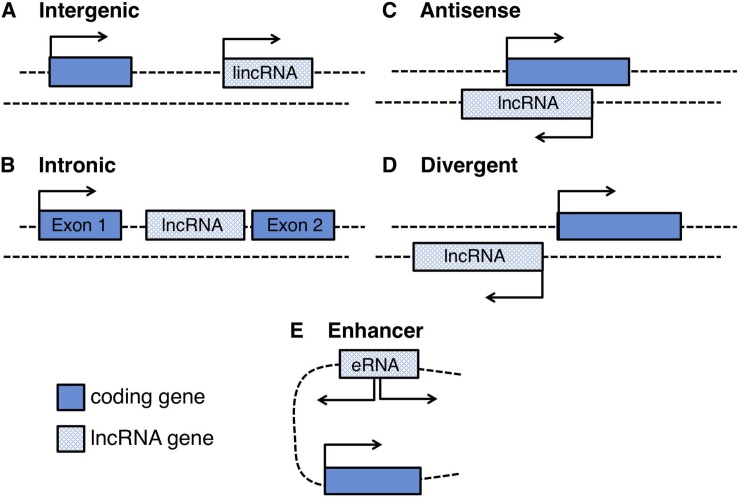
Schematic representation of the genomic loci of different long non-coding RNAs: **(A)** intergenic RNAs (lincRNAs), located between two protein coding-genes, **(B)** intronic lncRNAs, located inside introns of protein coding-genes, **(C)** natural antisense lncRNAs (NATs), transcribed in an antisense orientation with respect to a protein coding-gene **(D)** divergent lncRNAs originated from bidirectional transcription of protein-coding genes, and **(E)** enhancer RNAs (eRNAs) transcribed from bidirectional transcription of enhancer regions.

Large scale studies from multiple sources of data such as DNAse hypersensitivity and chromatin state maps released from the ENCODE (Encyclopedia of DNA Elements) consortium, revealed that 93% of the human genome is actively transcribed and 39% consists of transcriptional units composed by promoter and poly(A) signals: strikingly only a little more than 1% is protein coding. Analysis on multiple human cell and tissue types confirmed that lncRNAs largely outnumber the coding elements, are highly expressed in the nervous system and although their gene body is poorly conserved, the promoter regions and their structural motifs show higher evolutionary constraints (Mercer et al., 2008; Guttman et al., 2009; Derrien et al., 2012, 2014; Hon et al., 2017). Ambitious projects for the functional annotation of the mammalian genome (FANTOM) confirmed that lncRNAs are pervasively transcribed, producing a comprehensive understanding on the genomic organization of more than 50,000 lncRNA loci. Capped Analysis of Gene Expression (CAGE) linked with computational analyses shows that lncRNAs have a heterogeneous genomic organization and can be found: (i) as independent transcriptional units (intergenic RNAs or lincRNAs); (ii) transcribed divergent from coding genes thus sharing the same promoter (divergent lncRNAs); (iii) transcribed from intronic regions (intronic lncRNAs) or enhancer regions (eRNAs); (iv) transcribed as antisense RNAs with respect to coding genes (natural antisense transcripts, NATs) (Figure 1). Notably, around 70% of the mammalian coding genes show evidence of antisense transcription, producing ncRNAs that partially or completely overlap with their sense coding strand, their promoter or their regulatory regions (Zhang et al., 2006; Wanowska et al., 2018). Upon evidence of such widespread presence, the importance of NATs and their regulatory relationship with their sense counterparts were deeply dissected for many disease-associated genes and were shown to be particularly relevant in neurodegeneration and for the repeat expansion phenomenon, as it will be described in the following paragraphs.

## CircRNAs: Identification and Biogenesis

**FIGURE 2:**
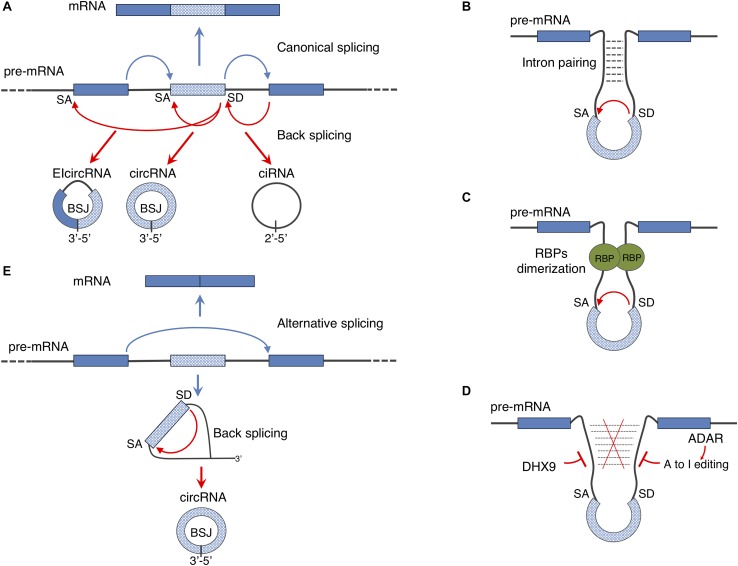
Regulation of circular RNA biogenesis: **(A)** canonical splicing generates mature mRNA while back-splicing can give rise to different kinds of circular transcripts: circRNAs can derive from exonic regions (circRNA), from introns (ciRNA) or from both exons and introns (ElcircRNA). **(B,C)** Back-splicing mechanism can be driven by intron pairing or RNA binding proteins (RBPs) dimerization. **(D)** Inhibitory activity of ADAR and DHX9 enzymes on back-splicing driven by intron pairing. **(E)** As a consequence of alternative splicing events, lariat containing the skipped exon can be re-spliced producing a mature circRNA. Through this mechanism both mature mRNA and a circRNA can be produced from a single pre-mRNA.

The identification of circRNAs in mammals happened in a serendipitous manner when RNA-seq of libraries prepared from ribosome-depleted RNA were computed in order to map non-canonical RNAs derived from genome rearrangements. The work by Salzman et al. (2012) led to the identification of a class of transcripts derived from coding loci and made by exons joint in a reverse order with respect to the one encoded in the genome. These transcripts were demonstrated to be circular in shape and to derive form a non-canonical splicing event named back-splicing (Salzman et al., 2012; Jeck et al., 2013); indeed, as a consequence of this event, circRNAs result to contain a downstream splice donor joint to an upstream splice acceptor (the back-splice junction, BSJ) (Figure 2A). Nowadays, the standard procedure adopted in order to identify circRNAs is the high depth RNA-seq of Ribosomal RNA depleted samples (Glazar et al., 2014). Eventually, the addition of an exonuclease treatment (for instance RNAse R) or poly(A) plus selection limits the presence of linear RNAs thus improving the sequence coverage of circRNAs (Jeck et al., 2013; Panda et al., 2017). Moreover, several pipelines have been developed so far in order to compute the RNA-seq datasets for identifying circRNAs, and is worth mentioning: find_circ (Memczak et al., 2013), CIRCexplorer (Zhang et al., 2014), circBase (Glazar et al., 2014), circRNA_finder (Westholm et al., 2014), and CIRI2 (Hansen, 2018). Since these algorithms differ significantly in the pool of circRNA species they predict, it is recommended to use at least two independent algorithms to ensure proper annotation of the BSJs (Szabo and Salzman, 2016).

Two elements have been described, so far, to facilitate back-splicing: intronic *cis*-elements and/or protein factors acting *in trans* (Figures 2B,C).

Analyses of the intronic sequences of genes producing circRNAs revealed that introns flanking circularizing exons are longer than the average and often contain complementary inverted repeats (Jeck and Sharpless, 2014; Liang and Wilusz, 2014; Westholm et al., 2014; Zhang et al., 2014). In humans the repetitive elements are frequently represented by ALU sequences (Jeck et al., 2013; Ivanov et al., 2015) and recent studies showed that the circularization of such exons is affected by the activity of the exonucleases DHX9 and of the editing enzyme ADAR (Rybak-Wolf et al., 2015; Aktaş et al., 2017) both interfering with intron pairing (Figure 2D). Notably, a global decrease in ADAR mediating editing of ALU sequences has been observed during the differentiation of human embryonic stem cells toward the neuronal fate (Osenberg et al., 2010); this might explain the overall upregulation of circRNA production occurring during neuronal differentiation observed in both flies and mammals (Rybak-Wolf et al., 2015).

Nevertheless, the presence of intronic repeats *per se* is not sufficient to explain the dynamic and tissue specific expression of circRNAs, that instead relies on the activity of RNA binding proteins (RBPs). In most of the cases, RBPs bind introns in a sequence specific manner and, through dimerization, promote the back-splicing reaction (Figure 2C). Muscle blind (MBL) in Drosophila, Quaking (QKI) and Fused in Sarcoma (FUS) in mammals as well as multiple hnRNPs (heterogeneous nuclear ribonucleoproteins) and SR (serine-arginine) proteins are directly involved in facilitating circRNA biogenesis (Ashwal-Fluss et al., 2014; Conn et al., 2015; Kramer et al., 2015; Errichelli et al., 2017; Fei et al., 2017).

It has been also demonstrated that the back-splicing reaction can be further regulated by exon skipping events: the lariat containing the skipped exon can be re-spliced thereby producing a mature circRNA (Figure 2E) (Barrett et al., 2015). This mechanism allows the generation of both linear and circular RNAs from a single precursor transcript.

Finally, intron lariats that escape debranching can give rise to a different class of circRNAs, named ciRNAs (circular intronic circRNAs) (Figure 2A). Even though, the molecular mechanisms of ciRNA biogenesis is still unknown it has been shown that a consensus RNA motif near the 5′ splice site confers intron lariats the resistance to the debranching activity (Zhang et al., 2013).

## Long Non-Coding RNA Functions

At the beginning of the post-genomic era scientists realized that the genome is pervasively transcribed (Lander et al., 2001). By that time, the pioneering studies on H19 and Xist were the only few examples showing the functional role of lncRNAs on imprinting (Bartolomei et al., 1991; Brown et al., 1991). However, the remainder of the full plethora of ncRNAs, their transcriptional significance and functional role, remained controversial for a long time. It was only after RNA-seq techniques were fully available that we could appreciate innovative studies that explored and characterized lncRNA molecular functions in different cellular and molecular contexts. For instance, the studies on MEG3, MALAT1, HOTAIR, and linc-MD1 have revealed the critical and versatile role of lncRNAs in shaping the complex mammalian regulatory networks, through different mechanisms of action (Rinn et al., 2007; Tripathi et al., 2010; Cesana et al., 2011).

Structural features in the lncRNA sequence play a key role in the assembly and regulation of multi-molecular complexes, by controlling the affinity for DNA, RNA, and proteins (Wang and Chang, 2011; Fatica and Bozzoni, 2014). It has been shown that, due to the lack of a functional ORF, the poor conservation of lncRNA molecules only reflects the lower sequence constraint and that, instead, conservation of their secondary structures is important for maintaining the functionality of these molecules (Smith et al., 2013; Derrien et al., 2014). On the other hand, lncRNAs show high sequence conservation in their promoter region where binding sites for important regulatory transcription factors allow their tissue-specific expression patterns (Guttman et al., 2009). Indeed, in human and murine embryonic stem cells 60% of lncRNAs identified are divergently transcribed with respect to coding genes and share the same promoter, leading to a coordinated expression of coding and non-coding transcripts during development and differentiation (Sigova et al., 2013).

LncRNAs can be localized in the nucleus, in the cytoplasm and even in or with the mitochondria, and their localization may anticipate their mode of action (Rackham et al., 2011; Cabili et al., 2015; Leucci et al., 2016).

**FIGURE 3:**
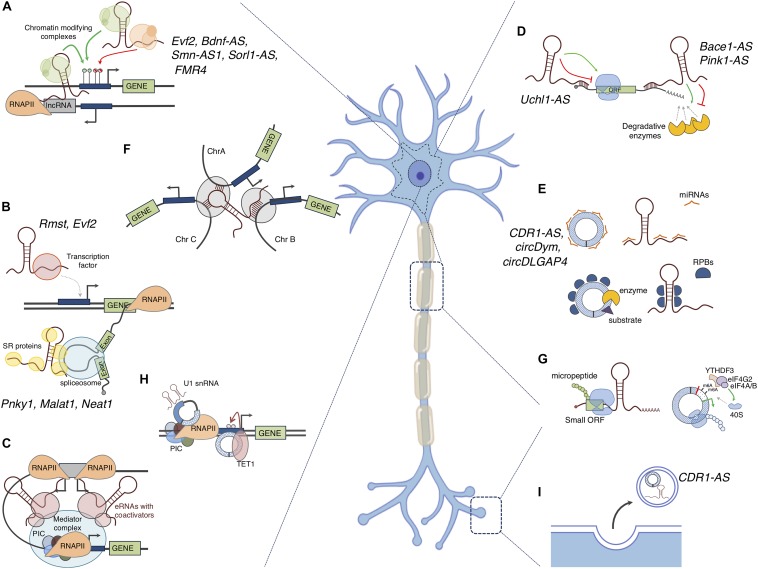
Mode of action of non-coding RNAs: The figure depicts the putative functions ncRNAs in the nucleus and in the cytoplasm of a neuron. **(A)** LncRNAs can act as scaffolds for recruiting chromatin-modifying complexes to gene promoters, thus silencing or inducing gene expression; *cis-* and *trans-* active mechanisms are shown; **(B)** LncRNAs can influence transcription of specific genes by acting as molecular decoys for transcription factors or can induce preferential inclusion or exclusion of exons, thus affecting the mRNA splicing patterns. **(C)** eRNAs can recruit transcriptional activators to distant promoters to activate gene expression; **(D)** LncRNAs can bind to mRNAs thus increasing their stability or inducing their decay. Induction or inhibition of translation is another outcome of the binding of lncRNAs to mRNAs. **(E)** CircRNAs and lncRNAs can interact with microRNAs (miRNAs) and RNA binding proteins (RBPs) titrating them away from their physiological targets or delivering them to the cell periphery (such as synapses). As scaffolds, they can also favor the interaction between enzymes and substrates. **(F)** LncRNAs can participate in genome architecture by coordinating the expression of genes located on different chromosomes. **(G)** LncRNAs can contain a small ORF that can be translated into functional micropeptides. In addition, an ORF can be generated upon circularization of AUG-containing exons, in this case circRNAs are translated in a Cap-independent manner. This translation can occur in the neuronal body or at the periphery, thus contributing to the protein content in this subcellular compartment. **(H)** CircRNAs can regulate transcription of their host genes by interacting with the transcriptional machinery or by recruiting epigenetic factors (TET1); **(I)** LncRNAs and circRNAs can be encapsulated and secreted into extracellular vesicles, for instance in response to neuronal activity, and might act as signal molecules for cell–cell communication. The names of lncRNAs and circRNAs with known functions in the nervous systems are indicated.

Inside the nucleus the scaffolding property of lncRNAs allows to guide protein factors or complexes to specific genomic loci, thus regulating their transcription and maturation in a positive or negative manner (Engreitz et al., 2016; Morlando and Fatica, 2018) (Figures 3A–C). Among these lncRNAs are Xist (recruits PRC2 for H3K27me3 and RYBP-PRC1 for H2A ubiquitylation; Zhao et al., 2010; Tavares et al., 2012), HOTTIP (recruits WDR5/MLL complex for histone H3 lysine 4 trimethylation; Wang et al., 2011) and eRNAs (recruit transcription factors and RNAPII; Kim et al., 2010; Lai et al., 2013). Cytoplasmic lncRNAs regulate gene expression at post-transcriptional level recruiting the appropriate protein machineries affecting the stability (lncRNA TINCR; Kretz et al., 2013), decay (1/2-sbsRNAs lncRNA; Gong and Maquat, 2011), translational activation (lnc-31; Dimartino et al., 2018) and repression (linc-p21; Yoon et al., 2012) of mRNAs (Figure 3D). Notably, many evidence reports that NATs have an impact on the sense coding strand in the cytoplasm by using their sequence complementary in order to mask miRNA binding sites (BACE1-AS; Faghihi et al., 2008) or to influence translation (Uchl1-AS; Carrieri et al., 2012). LncRNAs can also act as decoy molecules, which may inactivate transcription factors in the nucleus or sequester miRNAs and RBPs in the cytoplasm thus preventing them to bind their natural targets (Jpx lncrna, Sun et al., 2013; linc-ROR, Wang et al., 2013; linc-MD1, Cesana et al., 2011; lncMyoD, Gong et al., 2015) (Figures 3B,E).

Moreover, mRNA processing can be modulated by lncRNAs (Romero-Barrios et al., 2018). NEAT1 and MALAT1 are two examples of lncRNAs able to regulate the splicing of specific pre-mRNAs by modulating the phosphorylation state of splicing factors (Tripathi et al., 2010; Cooper et al., 2014) (Figure 3B).

For nuclear lncRNAs, *cis-* and *trans-* regulatory mechanisms have been described. *Cis-*acting lncRNAs affect the expression of proximal loci; examples are the NATs that interfere with the expression of the antisense coding gene by repressing or promoting its expression. The *cis-*regulatory mechanism is mediated via NAT transcription *per se* or through RNA-RNA interactions with the overlapping transcript. In this latter case, splice sites can be masked leading to alternative splicing events or transcript availability can be reduced through nuclear retention (Wanowska et al., 2018). A very common mechanism of *cis*-activity of NATs is the recruitment of epigenetic machineries, like the PRC2 thus influencing the chromatin state of their antisense coding gene (Yu et al., 2008; Modarresi et al., 2012) (Figure 3A).

Differently, *trans*-active lncRNAs affect the expression of target genes that are located in different chromosomes (Chu et al., 2011; Vance et al., 2014) (Figure 3A).

In the last few years several evidence has emerged tightly linking transcription and the three-dimensional organization of the genome, so adding an additional layer of complexity in gene expression regulation (Mele and Rinn, 2016). However, the cause-consequence relationship between transcription and genome re-organization was not univocally determined (Osborne et al., 2004; Zink et al., 2004). Many lncRNAs might participate in genome architecture through the simple act of transcription, independently of the mature RNA product or of their generally low abundance and lack of sequence conservation (Hangauer et al., 2013; Ballarino et al., 2018). Moreover, some mature lncRNA transcripts, such as XIST, FIRRE, and NEAT1, are known to play a role in organizing nuclear architecture (Mao et al., 2011; Engreitz et al., 2013; Hacisuleyman et al., 2014) (Figure 3F).

Ultimately, many apparent non-coding transcripts were revelaled to be associated to the ribosome and to produce small peptides (Hube and Francastel, 2018) (Figure 3G). The non-coding definition of lncRNAs relies on the arbitrary threshold of an ORF shorter than 100 aminoacids (Carninci et al., 2005); however, ribosome profiling revealed that translation is more pervasive than previously thought (Ingolia, 2014). Examples of lncRNA-derived micropeptides are myoregulin (encoded by the LINC00948 RNA in humans and by the 2310015B20Rik RNA in mice) and DWORF (encoded by LOC100507537 gene in humans and by NONMMUG026737 gene in mice) which regulate muscle performance by affecting the activity of the key calcium pump SERCA in a negative and positive manner respectively (Anderson et al., 2015; Nelson et al., 2016). Notably, DWORF may be useful therapeutic molecule in improving the cardiac muscle function of mammals with heart disease (Nelson et al., 2016). Ribosome associated lncRNAs have also displayed a dual function both as peptide coding and as ncRNA and are now termed bifunctional (Li et al., 2017). The lncRNA Oskar, involved in oocyte development is a prototypical example in this expanding research area (Jenny et al., 2006).

## Circular RNA Functions

The majority of circRNAs identified shows cytoplasmic localization even though there are some examples of nuclear localized molecules (Salzman et al., 2012; Jeck et al., 2013; Li et al., 2015a; Errichelli et al., 2017; Chen et al., 2018a). In these two compartments circRNAs control gene expression at different levels through mechanisms that have not been fully characterized.

Indeed, the function of few circRNAs has been unveiled so far. Several studies reported circRNAs exerting miRNA “sponge” activity, thereby altering the expression of the miRNA targets (Hansen et al., 2013; Memczak et al., 2013; Zheng et al., 2016; Bach et al., 2019) (Figure 3E). The most notable cases are the mouse Sry and the human CDR1-AS (also known as ciRS-7) which respectively, possess 16 binding sites for miR-138 and more than 70 evolutionary conserved binding sites for miR-7 (Hansen et al., 2013; Memczak et al., 2013). In particular CDR1-AS is expressed much more than any other housekeeping gene in mouse and human brain and this, together with the high number of miR-7 binding sites, makes the competing activity of CDR1-AS stoichiometrically relevant in neuronal tissue (Hansen et al., 2013). More recently, Piwecka et al. (2017) showed that CDR1-AS may modulate the activity of miR-7 by acting on its stability. Consistently with these data, the depletion of CDR1-AS causes the modulation of miR-7 targets mRNAs both *in vivo* and *in vitro* (Hansen et al., 2013; Memczak et al., 2013).

In addition to miRNAs, circRNAs can interact with RBPs. They may sequester them from other targets or regulate their stability/activity (Ashwal-Fluss et al., 2014; Du et al., 2017; Liu et al., 2019; Rossi et al., 2019); circRNAs may also act as molecular scaffolds to allow enzymes and substrates to become adjacent (Du et al., 2017; Zeng et al., 2017) (Figure 3E).

Examples of circRNAs containing an ORF, thus serving as templates for translation, have also been described (Legnini et al., 2017; Pamudurti et al., 2017; Yang et al., 2018; Zhang et al., 2018a; Liang et al., 2019). Given their circular structure, the translation of these circRNAs relies on a Cap-independent mechanism and on the presence of the N6-methyl-adenosine modification. This latter promotes the binding of the reader protein YTHDF3 and the IRES-specialized translation initiation factor eIF4G2 (Legnini et al., 2017; Yang et al., 2017) (Figure 3G).

In the nucleus, circRNAs can participate in gene expression regulation at transcriptional level. For instance, circPAIP2 and FECR1 circRNA have been described to regulate the transcription of their parental genes through two diverse mechanisms: interacting with transcription machinery, whilst the latter by recruiting the TET1 DNA demethylase (Li et al., 2015a; Chen et al., 2018a) (Figure 3H).

## Role of Non-Coding RNAs in the Physiology and Development of the Nervous System

LncRNAs show a crucial role in many stages of neuronal differentiation and specification by participating in gene expression control at the epigenetic, transcriptional, and post-transcriptional levels. LncRNA molecules may regulate the exit from the pluripotency state, influence cell fate choice during neural development and contribute to the synaptic activity in mature cells (Zalfa et al., 2003; Zhong et al., 2009; Guttman et al., 2011; Ng et al., 2012; Vance et al., 2014). The lncRNA mechanism of action is strictly linked to their secondary structure and their scaffolding activity, which translates into the ability to regulate gene expression by binding and recruiting secondary factors onto regulative domains. Here we report some examples that are noteworthy for the study of nervous system differentiation and in the maintenance of its physiological functionality (Table 1). RMST is a lncRNA controlled by the master regulator REST during neural differentiation (Ng et al., 2013). Depletion of RMST prevents cells from exiting the embryonic stem cell state and inhibits the initiation of neural differentiation. Indeed, RMST acts as a scaffold RNA by guiding the transcription factor Sox2 to the promoter of key neurogenesis-promoting genes, such as DLX1, ASCL1, HEY2, and SPS (Ng et al., 2013).

**TABLE 1:** List of ncRNAs with a known or potential function (asterisk) in neuronal physiology and disease.

**Name**	**Classification**	**Proximal/overlapping coding gene**	**Molecular function**	**Biological function**	**Disease**	**References**
RMST	lincRNA	N/A	Decoy for transcription factor	ESC self-renewal and inhibition of neural differentiation	N/A	Ng et al., 2013
Evf2	NAT	Dlx6	Scaffold RNA for transcription factors and chromatin remodeling	Neuronal differentiation and development	N/A	Bond et al., 2009
Pnky	Divergent	Pou3f2	Decoy for splicing factors	Neural Stem Cell self-renewal	N/A	Ramos et al., 2015
Cyrano	lincRNA	N/A	target RNA-directed miRNA degradation	ESC self-renewal	N/A	Kleaveland et al., 2018
Lhx1os	Divergent	Lhx1	Unknown	Neuronal Differentiation	ALS	Biscarini et al., 2018
LncMN-1	Divergent	Pcdh10	Unknown	Neuronal Differentiation	ALS	Biscarini et al., 2018
BC200	Intronic	Epcam-DT	Scaffold RNA for translation factors	Regulates translation at synapsis	AD	Muslimov et al., 1998; Wang et al., 2002; Zalfa et al., 2003
MALAT1	lincRNA	N/A	Decoy for splicing factors, paraspeckle	Neuronal Stress Response	ALS, HD, FTD, AD	Ji et al., 2003; Bernard et al., 2010
BDNF-AS	NAT	Bdnf	Scaffold RNA	Synaptogenesis	HD	Lipovich et al., 2012; Modarresi et al., 2012
SMN-AS1	NAT	Smn	Scaffold RNA	Neuronal Differentiation	SMA	d’Ydewalle et al., 2017
SORL1-AS	NAT	Sorl1	Scaffold RNA	Unknown	AD	Ciarlo et al., 2013
BACE1-AS	NAT	Bace1	RNA-RNA interaction for miRNA masking	Beta amyloid formation	AD	Faghihi et al., 2008; Faghihi et al., 2010
UCHL1-AS	NAT	Uchl1	RNA-RNA interaction through SINEUP	Neuronal Stress Response	AD, PD	Carrieri et al., 2015
NEAT1	lincRNA	N/A	Scaffold RNA for splicing factors, Paraspeckle	Neuronal Stress Response	ALS, HD, FTD, AD	Nishimoto et al., 2013; An et al., 2018
C9ORF72-AS	NAT	C9ORF72	RNA foci	Unknown	ALS	DeJesus-Hernandez et al., 2011; Cooper-Knock et al., 2015b
PINK1-AS	NAT	Pink1	RNA-RNA interaction in *cis* on Pink1	Mithocondrial function	PD	Scheele et al., 2007
FMR4	NAT	Fmr1	Scaffold RNA in trans for chromatin remodeling	Neural precursor proliferation	FXS, FXTAS	Khalil et al., 2008; Peschansky et al., 2016
HTT-AS	NAT	Htt	Decoy for transcription factor in *cis* on HTT	Unknown	HD	Chung et al., 2011
ATXN8-OS	NAT	Sca8	RNA foci	Unknown	SCA	Moseley et al., 2006
SCAANT1	NAT	Sca7	Decoy for transcription factor in *cis* on Sca7	Unknown	SCA	Sopher et al., 2011
CDR1-AS	circRNA	CDR1	miRNA sponge	Synaptic transmission	AD, PD	Hansen et al., 2013; Memczak et al., 2013; Piwecka et al., 2017
circRims2	circRNA	Rims2	Unknown	Unknown	N/A	Rybak-Wolf et al., 2015
circDym	circRNA	Dym	miRNA sponge	Microglial activation	Depressive-like behavior	Rybak-Wolf et al., 2015; Zhang et al., 2018b
circPldxnd1	circRNA	Pldxnd1	Unknown	Unknown	N/A	Rybak-Wolf et al., 2015
circStau2	circRNA	Stau2	Unknown	Unknown	N/A	Rybak-Wolf et al., 2015
circHomer1_a	circRNA	Homer1	Unknown	Homeostatic synaptic plasticity*	N/A	You et al., 2015
mmu_circRNA_017963	circRNA	Tbc1d30	miRNA sponge*	Apoptotic process, transport and RNA splicing, synaptic functions*	AD	Huang et al., 2018
circDLGAP4	circRNA	Dlgap4	miRNA sponge	Cell viability, apoptosis, mitochondrial damage, and autophagy	PD	Feng et al., 2019

*N/A is for “Not Applicable.”*

Evf2 lncRNA also regulates cell-fate choice and is a clear example of how RNA secondary structure may contribute through a variety of modes of action. This lncRNA is a NAT to the DLX6 gene and recruits the DLX transcription factor and the methyl-CpG-binding protein (MECP2) to the promoters of interneuron lineage genes (DLX5, DLX6, and GAD1) through both *cis-* and *trans-* acting scaffolding mechanisms, ultimately controlling the excitatory-inhibitory neurons balance *in vivo* (Bond et al., 2009). In addition, the Evf2 transcript is implicated in controlling the methylation state of DLX5/6e enhancer through a chromosomal looping mechanism, thus regulating the expression of DLX5/6 locus (Berghoff et al., 2013). However, how the Evf2 methylation control is combined with the recruitment of DLX and MECP2 is still unknown.

Pnky is a lncRNA that is few kilobases divergent from the Pou3f2 gene. This lncRNA, whose expression peaks in dividing neural stem cells (NSCs) and decreases during neuronal differentiation, is essential for self-renewal of the NSC population. Pnky interacts with the splicing regulator PTBP1 and controls the expression and alternative splicing of a core set of targets involved in neurogenesis (Ramos et al., 2015). Through this mechanism Pnky regulates the balance between self-renewal and differentiation of NSCs.

An interesting variation of this mode of action has been described for the lncRNA Cyrano that is highly expressed in the nervous system and recently shown to be implicated in a whole non-coding regulatory circuitry (Kleaveland et al., 2018). Kleaveland et al. (2018) identified a post-transcriptional regulatory network in which Cyrano binds miR-7, leading to its degradation through a target RNA-directed miRNA degradation mechanism (TDMD). Mir-7 degradation in nervous tissue blocks the repression of its RNA targets among which the circRNA CDR1-AS. Notably, Cyrano knock-down in zebrafish causes a neurodevelopmental phenotype (Ulitsky et al., 2011).

LncRNAs specifically expressed in *in vitro* derived murine motor neurons have also been identified (Biscarini et al., 2018). Two interesting cases are Lhx1os and lncMN-1 that are divergent from Lhx1 and Pcdh10 protein-coding genes respectively. Lhx1 encodes for a morphogenetic factor of the LIM family involved in lateral motor neuron differentiation, head development and motor neuron axon guidance, while Pcdh10 codes for a protocadherin involved in motor neuronal cell adhesion (Tsuchida et al., 1994; Hunter and Rhodes, 2005; Machado et al., 2014). Notably, Lhx1os and lncMN-1 show strong co-regulation with two divergent coding transcripts in both mouse and human motor neurons.

LncRNAs were shown to participate in mature neuron plasticity and physiology. Indeed, despite being post-mitotic cells, neurons need to maintain a high level of plasticity in order to be able to respond to stimuli and to re-arrange their synaptic network for accompanying processes such as learning, memory, cognition and recovery from injury or insult (Costa-Mattioli et al., 2009; West and Greenberg, 2011). The lncRNA BC1/BC200 regulates synaptogenesis. This transcript is expressed in the developing and adult nervous system where is transported to dendrites (Muslimov et al., 1997). In these cellular structures BC1/BC200 interacts with FMRP and the translational machinery in order to control the formation of the 48S complex leading to a repression of local translation at synapses (Wang et al., 2002; Zalfa et al., 2003). Moreover, the expression of BC1/200 is dynamically upregulated at specific synapses in response to neuronal activity, thus shaping the synaptic protein content (Muslimov et al., 1998).

MALAT1, initially characterized for its role in cancer metastasis, was then identified to have a role in synapse formation (Ji et al., 2003). This lncRNA is highly expressed in neurons and it is localized in nuclear speckles; MALAT1 controls the processing of synaptogenesis-related genes through the recruitment of SR-protein family members to transcription sites (Bernard et al., 2010).

Finally, some interesting mechanisms involving NATs have been shown to contribute to the regulation of neuronal plasticity. BDNF-AS, whose expression is dependent on neural activity also contributes to the decision between synaptic maintenance or elimination in response to the levels of stimulation by controlling the expression of its sense overlapping gene BDNF (Lipovich et al., 2012; Modarresi et al., 2012).

Similarly to lncRNAs, circRNAs have been recognized to play important roles in development and function of neuronal tissue (van Rossum et al., 2016) (Table 1). Recent studies have demonstrated that circRNAs are specifically enriched in brain tissue and in particular, they revealed that compared to other tissues, the mammalian brain contains the highest percentage of genes hosting circRNAs and that these genes hold the ability to produce the greatest number of distinct circRNAs (Rybak-Wolf et al., 2015).

Notably, neuronal circRNAs resulted to be regulated during embryonic development and conserved between rodents, pigs and humans (Westholm et al., 2014; Rybak-Wolf et al., 2015; Venø et al., 2015; Chen et al., 2018b). In a model system of neuronal differentiation the overall expression of circRNAs resulted to be significantly upregulated and gene ontologies of their host genes showed their enrichment in neuronal specific pathways such as neuron development, differentiation, and synaptic transmission (You et al., 2015). Other studies further showed that circRNAs result differentially expressed in various anatomic brain regions and at specific embryonic or postnatal stages (Rybak-Wolf et al., 2015; Venø et al., 2015). Moreover, based on differential expression between adult and aged brains, these studies also showed that the expression of specific circRNAs is aging-related (Westholm et al., 2014; Gruner et al., 2016; Xu et al., 2018). Some examples are the murine circRims2 and circDym which are expressed more than 50% in adult cerebellum and circPldxnd1 which instead is predominantly expressed in prefrontal cortex (>60%) with respect to the other brain regions (Rybak-Wolf et al., 2015). In addition, Venø et al. (2015) found that during the porcine embryonic brain development the expression of clusters of specific circRNAs coincides with distinct developmental transitions and that the maximum expression and complexity of circRNAs is reached at a time corresponding to the period of major neurogenesis (form day 48 to day 60). Finally, it has been also demonstrated that circRNA expression is modulated during *in vitro* differentiation of murine motor neurons with specific circRNAs exclusively expressed in this cell type (Errichelli et al., 2017).

All these data strongly support the idea that circRNAs play important biological functions during development and specification of the nervous system.

Besides the expression modulation, neuronal circRNAs also show an intriguing subcellular localization since they have been found to be enriched in synaptoneurosomes more than their linear counterparts (Rybak-Wolf et al., 2015; Venø et al., 2015; You et al., 2015). An example is circStau2 that is mainly located at synapses while the linear Stau2 is primarily cytoplasmic (Rybak-Wolf et al., 2015). Furthermore, the most abundant neuronal circRNAs derive from genes encoding for proteins associated with synaptic functions (Venø et al., 2015; You et al., 2015). In this regard, You et al. (2015) demonstrated that circHomer1_a, which originates from the Homer1 gene encoding for a key protein in post-synaptic density regulation, reaches its highest expression and synaptic localization during synaptic plasticity in cultured hippocampal neurons (Meyer et al., 2014; You et al., 2015). In the same study, You et al. (2015) also analyzed the expression of circRNAs at various stages of brain development in mice (from E18 to P30) and showed that in hippocampal neurons an abrupt postnatal shift in circRNA expression (and not of the linear host transcripts) occurs when synaptogenesis begins. Lastly, mice carrying a deletion of the CDR1-AS genomic region exhibited defects in excitatory synaptic transmission and impaired sensorimotor gating; an increased spontaneous vesicle release was also observed in the neurons of these mice, suggesting that CRD1-AS might have a role in regulating synaptic transmission (Piwecka et al., 2017).

Even though, the molecular mechanism allowing the circRNAs delivery at the neuronal periphery is still unknown, all the findings suggest a role for these RNAs in the formation and activity of specific neuronal structures: circRNAs might be selectively transported to synapses to regulate their activity functioning as sponges/cargo for miRNAs and RBPs (Figure 3E). In this way they might indirectly regulate the expression of the miRNA/RBP-targeted mRNAs at synapses. Alternatively, synaptic circRNAs might function as signal molecules since they can also be packaged into vesicles and released into the synaptic cleft to influence neighboring cells (Li et al., 2015b; Lu and Xu, 2016) (Figure 3I).

## Long Non-Coding RNAs in Neurodegenerative Diseases

Alteration of lncRNA expression has been extensively described for many neuronal diseases together with their wide implication in the formation of aberrant molecular pathways (Salta and De Strooper, 2017). In addition, among the non-coding genes, lncRNAs are highly expressed in the nervous system and have been often observed to be located in proximity to neuronal genes and loci associated with neurodegenerative diseases (Qureshi et al., 2010; Wei et al., 2018). Starting from their genomic localization (intergenic, antisense, intronic, etc.) researchers focused on understanding the role that these molecules may play in *cis* or *trans* to control gene expression. As described above, several mechanisms of action have been observed and here we report some evidence where lncRNA activity is altered in neurodegenerative diseases (Table 1). Numerous examples are of NATs involved in neuronal processes and associated with neurodegeneration. NATs can regulate gene expression by recruiting chromatin modifiers (i.e., SMN-AS1) or by impacting the splicing of the sense coding-strand (i.e., SORL1-AS). Other lncRNAs work independently from their neighboring genes and may act as scaffolds in specific stress related paraspeckles (i.e., NEAT1), rather than regulate mRNA stability by forming RNA-RNA interactions (i.e., BACE1-AS, UCHL1-AS, PINK1-AS).

In spinal muscular atrophy (SMA) mutations in the SMN1 gene, encoding for the survival motor neuron protein (SMN), is the cause of the disease. In addition to SMN1 gene, SMN protein can be also produced by a splicing variant of SMN2, a gene present in variable copies for every individual. When mutations of SMN1 occur, depending on SMN2 copy number, SMA develops showing diverse clinical severities (Gavrilov et al., 1998). d’Ydewalle et al. (2017) identified a SMN antisense transcript (SMN-AS1), whose expression levels increased in neuronal differentiation, inversely correlating with the SMN protein. They found that SMN-AS1 recruits the chromatin modifier PRC2 to the SMN2 promoter repressing its gene expression. SMN-AS1 knock-down dissociates PRC2 from the promoter, thus increasing the overall SMN protein levels in neurons. This indicates that the levels of SMN-AS1 play an important role in the balance of residual SMN protein, thus impacting on the clinical outcome of the SMA disease (d’Ydewalle et al., 2017).

SORL1 antisense RNA (SORL1-AS) is produced from the Sortilin Related Receptor 1 (SORL1), a gene involved in amyloid-β formation in neuronal cells (Massone et al., 2011). Observations in post-mortem cerebral cortices of Alzheimer disease (AD) compared to healthy individuals showed that SORL1 levels increase and inversely correlate with SORL1-AS (Ciarlo et al., 2013). Ciarlo et al. (2013) found that SORL1-AS expression drives a splicing shift of SORL1 from the synthesis of the canonical long protein variant A to an alternatively spliced protein isoform. This process, resulting in a decreased synthesis of SORL1 variant A, is associated with impaired processing of amyloid precursor protein (APP) leading to increased amyloid β formation. The level and the activity of SORL1-AS in the etiology of the disease becomes crucial and may represent an interesting target for future therapeutic strategies (Ciarlo et al., 2013).

NEAT1 is a mammalian lncRNA that is ubiquitously expressed and has a scaffold role in the formation of subnuclear bodies termed paraspeckles. It presents two major isoforms, a 3.7 kb NEAT1_1 and 23 kb NEAT1_2 (Hutchinson et al., 2007). Nishimoto et al. (2013) observed NEAT1_2 to be upregulated during the early stages of amyotrophic lateral sclerosis (ALS) pathogenesis and found it to be present in paraspeckles of ALS patients, providing, for the first time, a direct association between paraspeckle formation and neurodegenerative disease (Nishimoto et al., 2013). Paraspeckles are stress-responsive nuclear bodies, which increase in size and number and accompany several physiological as well as pathological stressful conditions (An et al., 2018). Mechanistically, increased paraspeckle formation is observed in ALS and is accompanied by nuclear depletion of TDP-43, a protein frequently dysregulated in ALS (Mackenzie et al., 2010). Indeed, TDP-43 binds NEAT1_2, and when mutated or downregulated affects NEAT1_2 accumulation and paraspeckle assembly (Nishimoto et al., 2013). In Huntington disease (HD), frontotemporal dementia (FTD), and AD an increase of NEAT1_2 was observed in patient cohorts and experimental studies suggest that NEAT1_2 fine-tunes the function of multiple neurodegeneration-associated pathways, like mitochondrial signaling and miRNA biogenesis (An et al., 2018).

BACE1-AS is an antisense transcript originating from the BACE1 (β secretase 1) gene coding for a protein which is essential for the generation of β-amyloids. This lncRNA is evolutionarily conserved across vertebrates and was observed to be elevated in subjects with AD and in APP transgenic mice (Faghihi et al., 2008). BACE1-AS has an important role in enhancing the stability of BACE1 mRNA via the formation of a RNA duplex, thus masking miR-485-5p binding sites and leading to an increase of BACE1 protein (Faghihi et al., 2010). The knock-down of this antisense transcript decreases the level of BACE1, reducing amyloid formation and aggregation in the brain. BACE1-AS represents a clear biomarker and potential therapeutic target for the treatment of AD.

Another antisense RNA is UCHL1-AS (ubiquitin carboxy-terminal hydrolase L1-antisense) associated with AD, whose activity depends on the presence of a 5′ overlapping sequence with UCHL1 and an embedded inverted SINEUP (SINEB2 sequence to UP-regulate translation). Thanks to the formation of the RNA-RNA duplex with UCHL1 mRNA, UCHL1-AS enhances Cap-independent UCHL1 protein translation under stress condition (Carrieri et al., 2012). UCHL1 expression is associated with a delay of AD, making UCHL1-AS a fundamental regulator of the disease onset and a promising target for therapeutic intervention. Interestingly, both UCHL1 and UCHL1-AS are also found to be downregulated in Parkinson’s disease (PD) (Carrieri et al., 2015).

Another antisense transcript, PINK1-AS, is transcribed from the antisense direction of the PINK1 gene (PTEN phosphatase and tensin homolog deleted on chromosome 10-induced putative kinase 1), a gene abundant in mitochondrial-rich tissues and often mutated in PD (Gispert et al., 2009). PINK1-AS controls *in cis* the expression of a PINK1 splice variant, the svPINK1 transcript, through the formation of a RNA-RNA duplex. The silencing of PINK1-AS results in the reduced expression of svPINK1 in neuronal cells (Scheele et al., 2007). Given that, svPINK1 codes for a homolog of the C-terminus of PINK1, a peptide sequence which regulates PINK1 kinase activity; modulation of PINK1-AS expression may therefore have direct relevance in PD.

Given these examples, it is of no surprise that lncRNAs may play crucial roles in many other molecular processes involved in neurodegeneration. Importantly, except for few reports, most of these molecules are located in proximity or antisense to critical neuronal loci and so the dissection of these specific classes of molecules requires specific attention.

## Antisense Transcription of Nucleotide Repeat Expansions Is Involved in Neurodegeneration

A conspicuous amount of evidence coming from the transcription of nucleotide expansions emphasizes a role of NATs alongside the coding sense strand in the etiology of neurodegenerative diseases (Salta and De Strooper, 2017; Zucchelli et al., 2019). Here, we describe how nucleotide repeat expansion-associated NATs impact on the disease through complex gain- and loss- of-function mechanisms. One well-exemplified case is described for C9ORF72, a gene that harbors a hexanucleotide repeat expansion representing the most common cause of FTD and ALS. In the mutated loci the sense strand codes for an ORF that produces a repetition of six dipeptide proteins (RAN) forming co-aggregates in the cytoplasm of neurons (DeJesus-Hernandez et al., 2011). This locus is also transcribed in the antisense direction and both the sense/antisense RNA transcripts accumulate to form disease-associated nuclear RNA foci, the number of which correlates with the clinical severity of ALS and FTD (DeJesus-Hernandez et al., 2011; Cooper-Knock et al., 2015a). Interestingly, the knock-down of sense expanded C9ORF72 transcripts through the use of antisense single-stranded oligonucleotides (ASOs) in *in vitro* derived motor neurons mitigates defects in nucleocytoplasmic transport and glutamate toxicity phenotype, but is not sufficient to fully revert the molecular signatures derived from the hexanucleotide expansion (Donnelly et al., 2013; Lagier-Tourenne et al., 2013; Zhang et al., 2015; Jiang et al., 2016). These findings strongly suggest that the antisense ncRNA and the associated RNA foci contribute to the neurodegenerative phenotype. Notably, antisense C9ORF72 RNA foci hijack RBPs as sense RNA foci but, differently from these, antisense foci are associated with TDP-43 mislocalization in motor neurons from C9ORF72 patients (Cooper-Knock et al., 2014, 2015b). All these findings point to the importance of targeting both sense and antisense expanded C9ORF2 transcripts in order to develop an effective therapeutic approach.

Fragile X mental retardation 1 (FMR1) locus is characterized by the production of multiple non-coding transcripts (FMR5, FMR6, FMR4) in addition to the FMR1 mRNA. Expansion of the CGG triplet in the FMR1 gene (>200 repeats for complete penetrance) is attributed as the main cause of Fragile X syndrome (FXS), and in a pre-mutation state (55–200 repeats) is responsible for Fragile X-Associated Tremor/Ataxia Syndrome (FXTAS) (Rajan-Babu et al., 2017). Although the pathogenic relevance of all the FMR1 associated transcripts remains to be fully defined, FMR4 is a lncRNA antisense to FMR1 that spans the repeated region and that was observed to significantly affect human cell proliferation and apoptosis *in vitro* (Khalil et al., 2008). Peschansky et al. (2016) confirmed the proliferative effect of FMR4 also in human neural precursor cells (hNPCs) and determined that this lncRNA alters the chromatin state of 100s of genes *in trans*, with a significant enrichment for genes involved in neural development and proliferation (Peschansky et al., 2016).

In HD expansion of a CAG-repeat in the huntingtin gene (HTT) results in an elongated polyglutamine stretch and is the main cause of the pathology. From the HTT locus, two ncRNAs are produced: a small CAG containing RNA (sCAG) of around 21 nts with neurotoxic Ago2-dependent activity and an antisense lncRNA (HTT-AS) overlapping with the repeated expansion and observed to be reduced in human HD frontal cortex (Chung et al., 2011; Bañez-Coronel et al., 2012). Additionally, HTT-AS acts as a transcriptional repressor of HTT gene, thus suggesting a protective role of this lncRNA in the penetrance of the disease (Chung et al., 2011).

In spinocerebellular ataxias (SCA) CAG expansions are found in several loci that code for poly-Q SCA proteins. In the SCA8 locus, an antisense transcript (ATXN8-OS/KHL1-AS) that includes the reverse complement of the expansion (CTG) is produced and accumulates in RNA foci in the brain (Moseley et al., 2006); in the SCA7 locus, convergent transcription is also found to produce an antisense SCAANT1 lncRNA. SCAANT1 suppresses SCA7 expression in mice and inversely correlates with SCA7 expression in SCA patients, thus suggesting a loss-of-function mechanism where the lncRNA is involved (Sopher et al., 2011).

Altogether these observations prove that NATs may impact on the penetrance and severity of the clinical symptoms of many neuronal diseases, thus re-centering the attention of the research on new therapeutic strategies and modes of intervention as described in the following paragraphs.

## Circular RNAs in Neurodegenerative Diseases

Recent investigations have suggested that circRNAs not only function in physiological conditions but that they may also play crucial roles in the occurrence and development of neurological diseases (Table 1).

The first evidence comes from the observation that deficiency or mutations in proteins involved in circRNA biogenesis are linked to the pathogenesis of neurodegenerative diseases: for instance deficiency of QKI may contribute to the development of inherited ataxia while mutations of the FUS gene as well as the deregulation of ADAR2 expression are linked to the pathogenesis of ALS (Chénard and Richard, 2008; Kwiatkowski et al., 2009; Vance et al., 2009; Hideyama et al., 2012; Aizawa et al., 2016).

The implication of circRNAs in neuronal diseases is further supported by studies of expression profiling performed in cellular or animal model systems and by using patients’ specimens. Regardless the different modes of action attributed to circRNAs, their activity as miRNA sponges is the only one explored in all the studies reporting implications of these ncRNAs in neuronal disorders. However, this evidence is far from considering the miRNA sponge activity as a general mode of action of this class of ncRNAs in neurons. Indeed, besides CDR1-AS, most of the circRNAs defined as sponges have only one or a very few binding sites for miRNAs, making the effectiveness of their sponge activity questionable (Li et al., 2019; Ragan et al., 2019). Nevertheless, it has been detected that subsets of circRNAs could act in concert to exert reasonable miRNA sponge function (Ragan et al., 2019).

Huang et al. (2018) have identified more than 300 circRNAs deregulated in the hippocampus of 5- and 10- month-old senescence-accelerated mice P8 (SAMP8), an AD animal model, compared to WT mice. Among them, they characterized mmu_circRNA_017963 circRNA, which might be involved in several cellular processes including apoptosis and synaptic function (Huang et al., 2018). Furthermore, microarray technology combined with RNA-seq analysis allowed to simultaneously characterize circRNA, miRNA and mRNA expression in the hippocampus of an AD rat model in order to build putative regulatory networks linked to AD pathogenesis (Wang et al., 2018b).

These analyses identified two possibly AD-linked networks involving the genes Iodothyronine Deiodinase 2 (Dio2) and the high-mobility group box 2 (HMGB2). In particular, the expression of Dio2, that activates myelination, and of HMGB2 which controls the amyloid-β plaque clearance, is altered in AD (Calza et al., 2002; Gao et al., 2011; Humphries et al., 2015; Yamanaka et al., 2015). The networks identified by *in-silico* analyses have linked the deregulation of these two genes to the aberrant expression of specific circRNAs acting as sponges of mir-122-5p for Dio2 and of let-7-g-3p for HMGB2.

Furthermore, the most expressed and studied circRNA, CDR1-AS, was found reduced in hippocampal CA1 samples from sporadic AD patients compared to controls (Lukiw, 2013). As aforementioned, CDR1-AS has multiple binding sites for miR-7 and its reduction in AD conditions has been hypothesized to increase the levels miR-7 which in turn targets the ubiquitin protein ligase A (UBE2A). Notably, UBE2A plays an essential function for the proteolytic clearance of amyloid-β peptides and its expression is indeed reduced by 2.8 folds in the hippocampal CA1 regions of AD brains (Zhao et al., 2016). Through a similar mechanism CDR1-AS could be also involved in PD since also α-synuclein is a target of miR-7 (Junn et al., 2009). Indeed, it has been demonstrated that the repression effect of miR-7 on α-synuclein expression in human cell lines can be rescued by the concomitant overexpression of CDR1-AS (Hansen et al., 2013).

CircDLGAP4, originally reported to ameliorate ischemic stroke outcomes, is found to be downregulated in both MPTP-induced PD mouse and MPPþ-induced PD cell models (Bai et al., 2018; Feng et al., 2019). Feng et al. (2019) demonstrated that circDLGAP4 participates in PD biological processes regulating miR-134-5p activity. The reduction of CircDLGAP4 expression in PD conditions allows miR-134-5p to repress CREB1 and, as a consequence, the CREB1 target genes including BDNF, Bcl-2 and PGC-1. This would contribute to the development of PD via affecting cell viability, apoptosis, mitochondrial damage and autophagy in human and mouse (Feng et al., 2019).

Deregulation of circRNAs and not of their linear counterparts, has also been reported in *in vitro* derived motor neurons lacking the FUS gene or carrying FUS mutations linked to a severe form of familial ALS (Errichelli et al., 2017; D’Ambra et al., 2019), suggesting a possible role of circRNAs in the pathogenesis of this disorder. In particular, Errichelli et al. (2017) demonstrated that FUS impacts directly on the biogenesis of specific circRNAs through the binding of intronic regions involved in circularization. Whether circRNAs deregulation is caused by loss or gain of function of FUS mutations still remains to be addressed.

## Non-Coding RNA Diagnostics and Therapeutics

One of the major challenges for researchers pursuing the understanding and ultimately the treatment of neurological disorders is early diagnosis. By this matter, it is of crucial importance to find suitable molecular markers detectable in patients’ specimens obtained through non-invasive methods. In this regards, liquid biopsies are the most applied non-invasive method to measure biomarkers that are soluble in body fluids such as plasma, blood, saliva, and cerebrospinal fluid (CSF). It is believed that circulating molecules potentially reflect the type of disease and can be detected at early stages when other diagnostic tools are not effective (Kelemen et al., 2019).

NcRNAs have been recognized as very important markers in the field of molecular diagnosis since they can be easily detected and quantified in body fluids. LncRNAs and even more circRNAs are high stable while circulating in body fluids, especially when included into extracellular vesicles (Figure 3I). Moreover, they may reflect the origin of the disease because of their tissue specificity. There are many examples of lncRNAs and circRNAs already proposed as possible diagnostic and prognostic biomarkers for various illnesses including cancer, diabetes, Crohn’s disease, coronary artery disease, and rheumatoid arthritis (Li et al., 2015b; Ouyang et al., 2017; Zhao et al., 2017; Kelemen et al., 2019). Nevertheless, few reports are available on lncRNAs and circRNAs as biomarkers for neurodegenerative diseases. In particular, Feng et al. (2018) studied the potential of selected lncRNAs as biomarkers in AD by analyzing plasma from a population of 88 AD patients vs. 72 control individuals. They found that BACE1 lncRNA may be a potential candidate biomarker to predict AD since it was significantly higher in AD patients than in healthy controls and showed high specificity (88%) for AD (Feng et al., 2018).

In another study the RNA extracted from CSF of a cohort of 27 PD patients and 30 controls was analyzed by RNA-seq: among the differentially expressed transcripts, the lncRNA SCN9 antisense (AC010127.3) and two lncRNAs close to LRRK2 locus (AC079630 and UC001lva.4) have been suggested as potential RNA biomarkers for diagnosis and response to treatment of PD (Hossein-Nezhad et al., 2016).

Moreover, Gagliardi et al. (2018) analyzed lncRNA expression in Peripheral Blood Mononuclear Cells of sporadic ALS and found 293 lncRNAs differentially expressed between normal control and sporadic ALS patients. Among these, NATs of genes which are already linked to neurodegenerative disease (Gagliardi et al., 2018).

As reported by this review, numerous studies have revealed a plethora of lncRNAs and circRNAs differentially expressed and whose activity is altered in disease conditions. This knowledge allows to identify RNA candidates to be used as markers for diagnosis and response to treatments, and even more importantly as potential therapeutic molecules. One interesting example comes from the work on UCHL1-AS and its functional role in up-regulating Uchl1 translation (Carrieri et al., 2015). Based on this study, Zucchelli et al. (2015) have designed synthetic SINEUPs to potentially target any mRNA in the cell. One application has been shown very recently in PD, where an increase of GDNF levels is beneficial for the reduction of the neurodegenerative symptoms. Previous therapeutic strategies to increase GDNF levels have produced side effects due to high ectopic doses of this factor (Kordower and Bjorklund, 2013). Using a PD mouse model and adeno associated viral (AAV) delivery of miniSINEUPs, a twofold increase of GDNF was observed in dopaminergic neurons, thus ameliorating motor deficits of the mice (Espinoza et al., 2020). MiniSINUPs are an encouraging approach for the increase of endogenous GDNF levels in patients and may represent a unique RNA-based therapeutic platform to address many other diseases.

A promising strategy to target ncRNAs takes advantage of the use of ASOs designed to bind perfectly to target transcripts, inducing either their enzymatic degradation or inhibition of the binding of RBPs required for RNA maturation/activity. It is likely that ASOs targeting NATs represents a powerful tool for novel therapeutic strategies, considering that NATs are pervasively associated with coding genes loci and have an impact on the regulation of neuronal genes. For instance, ASOs designed against BACE1-AS and SMN1-AS have been tested in murine and human model systems and have provided proofs of principle that these NATs are clinically relevant novel therapeutic targets for AD and SMA respectively. Notably, the downregulation of BACE1-AS in an AD mouse model lowers the amyloid-β levels and ameliorates adult neurogenesis while reduced levels of SMN1-AS increases the transcription of SMN2 gene in patient-derived cells, in SMA neurons, and in a mouse model of severe SMA (Modarresi et al., 2011; d’Ydewalle et al., 2017). As demonstrated in these two cases, the increase of knowledge concerning the biology of NATs in normal and disease states still represents the most important milestone to achieve in order to develop and design novel therapeutic approaches.

Another example of ncRNA used as potential therapeutic target is the repeat-containing C9orf72 transcript. A new approach that has been employed to knock-down these transcripts is the use of artificial miRNAs (miC). Notably, Martier et al. (2019) proved, *in vivo*, the delivery and efficacy of AAV5-miC in cortex and hippocampal neurons of Tg(C9orf72_3) ALS mouse model thus providing a proof of concept for the use of this strategy in the treatment of ALS and FTD.

Differently to lncRNAs, the field of circRNA research is still in its infancy and even though the use of these RNAs in the diagnosis and treatment of neurological disorders can be foreseen, we are still far from employing circRNAs in clinical practice.

Indeed, aside from the study by Cui et al. (2016), observing modulation of hsa_circRNA_103636 expression in peripheral blood mononuclear cells of patients with major depressive disorder treated with antidepressants for 8 weeks, the potential use of circRNAs as biomarkers in neurological diseases has not been well-explored yet. Additionally, the study by Cui et al. (2016) suggests that circulating circRNAs can be also used to assess responses to drug treatments.

The potential use of circRNAs in therapy comes from evidence suggesting that the accumulation of ciRNAs in the cytoplasm, caused by the inhibition of debranching enzyme 1 (Dbr1) activity, suppresses the toxicity of TDP-43 aggregates in human neuronal cell line and primary rat neurons (Armakola et al., 2012). ciRNAs might act as decoys for TDP-43 thus avoiding its interaction with other cellular RNAs in the cytoplasm. Since TDP-43 is deposited in protein aggregates in neurons and glia in > 96% of ALS cases, the modulation of ciRNAs biogenesis by targeting Dbr1 might represent a therapeutic strategy for ALS and other related TDP-43 proteinopathies.

Lastly, one important point of discussion arises for the delivery of therapeutic molecules to central nervous system (CNS) since the blood–brain barrier (BBB) and blood spinal cord barrier (BSCB) represents a bottleneck in the development of new therapies to treat CNS diseases. Indeed, in the last decade a great deal of effort has been dedicated to the achievement of an efficient and effective drug delivery to CNS focusing on the types of administration as well as on the design and modification of the potential therapeutic molecules (Krizbai et al., 2016; Kumar et al., 2017; Alexander et al., 2019; Fowler et al., 2020). A promising approach to circumvent the BBB and the BSCB is the delivery of therapeutic molecules directly to CNS through intrathecal injection (IT). Indeed, ASOs or AAV based molecules that have been IT administrated through intracerebroventricular (ICV) injection in rodent models and non-human primates, showed a widespread distribution in brain and spinal cord indicating the feasibility of this approach in targeting tissues mostly affected in neurodegenerative diseases (DeVos and Miller, 2013; Rigo et al., 2014; Biferi et al., 2017; Casaca-Carreira et al., 2017; Schoch and Miller, 2017; Martier et al., 2019). More importantly, pre-clinical and clinical trials involving IT/ICV delivery of ASOs against disease-associated transcripts (SMN, SOD1, and C9ORF72) have demonstrated the effectiveness and tolerability of this approach (Miller et al., 2013; Finkel et al., 2016; Cappella et al., 2019; Neil and Bisaccia, 2019).

## Perspectives

The knowledge derived from the studies on ncRNAs has increased exponentially in the last decade. Advances from international consortia, such as the FANTOM and the ENCODE projects for the functional identification of the whole transcriptome repertoire, have created a clear picture that extended regions of the genome are actively transcribed and contain previously undiscovered functional elements. Ambitious projects for the characterization of novel functions of non-coding transcripts and in particular of lncRNAs and circRNAs have deepened our understanding on the regulatory processes that underlie higher eukaryotes molecular complexity. This is particularly intriguing for the study of the nervous system, where tissue and cellular complexity seem to be evolutionary associated with an increase of non-coding genes number, expression and activity (Qureshi and Mehler, 2012). In this review we have described how lncRNAs and circRNAs are involved in controlling multiple neuronal functions in physiological as well as in pathological conditions. However, in this latter case most of the experimental studies focused on the differential expression of ncRNAs in disease respect to healthy conditions, while only partial information on ncRNA functions is available so far. Surprisingly, ncRNAs act through very diverse modes of action and, except few cases, no common feature is known to predict the function, making the study of each lncRNA or circRNA an incredibly challenging process.

The development of murine and cellular model systems, such as patient derived Induced Plutipotent Stem Cells efficiently differentiated through specific protocols, represent powerful model systems for the study of ncRNA functions in neurodegenerative diseases (Dawson et al., 2018; Wu et al., 2019). Indeed, these systems offer the opportunity to compare healthy with disease conditions providing mechanistic insights into molecular principles of neurodegenerative biology. The study of ncRNAs in these contexts might provide a unique resource for high-throughput functional screenings of non-coding genes involved in neurodegeneration.

Moreover, the use of model systems as well as patient specimens could represent a helpful resource for the identification of candidates having therapeutic potential, particularly in the preclinical stages when the neuronal loss is still minimal leading to a more effective intervention. As described in this review efforts to attain this goal have already started.

## Author Contributions

MM and BS wrote and revised the manuscript. SB prepared figures and revised the manuscript.

## Conflict of Interest

The authors declare that the research was conducted in the absence of any commercial or financial relationships that could be construed as a potential conflict of interest.
